# Analgesic Comparison of Flunixin Meglumine or Meloxicam for Soft-Tissue Surgery in Sheep: A Pilot Study

**DOI:** 10.3390/ani11020423

**Published:** 2021-02-06

**Authors:** Abbie V. Viscardi, Emily J. Reppert, Michael D. Kleinhenz, Payton Wise, Zhoumeng Lin, Shawnee Montgomery, Hayley Daniell, Andrew Curtis, Miriam Martin, Johann F. Coetzee

**Affiliations:** 1 Department of Anatomy and Physiology, College of Veterinary Medicine, Kansas State University, Manhattan, KS 66506, USA; paytonw@vet.k-state.edu (P.W.); zhoumeng@ksu.edu (Z.L.); smontgomery@vet.k-state.edu (S.M.); akcrz4@vet.k-state.edu (A.C.); miriammartin@vet.k-state.edu (M.M.); jcoetzee@vet.k-state.edu (J.F.C.); 2 Department of Clinical Sciences, College of Veterinary Medicine, Kansas State University, Manhattan, KS 66506, USA; erepper@vet.k-state.edu (E.J.R.); mkleinhe@vet.k-state.edu (M.D.K.); 3 Institute of Computational Comparative Medicine, College of Veterinary Medicine, Kansas State University, Manhattan, KS 66506, USA; 4 Animal Sciences and Industry, College of Agriculture, Kansas State University, Manhattan, KS 66506, USA; hdaniell@ksu.edu

**Keywords:** analgesia, animal welfare, flunixin meglumine, grimace scale, meloxicam, NSAID, ovine, pain, refinement, sheep

## Abstract

**Simple Summary:**

Pain management is lacking in U.S. commercial sheep production systems. This is, in part, due to the limited amount of scientific data evaluating sheep pain responses after analgesia treatment. Non-steroidal anti-inflammatory drugs (NSAIDs), such as meloxicam (MEL) and flunixin meglumine (FLU), are the most common drug class provided to livestock species to manage pain. Pain assessment tools, such as facial grimace scales, which use changes in facial expression to monitor pain, are also needed to improve pain management and sheep welfare. In this study, sheep undergoing a laparotomy (a surgical procedure where an incision is made into the abdominal cavity) were treated with either MEL or FLU to manage pain. A third group of ewes did not undergo surgery and served as study controls (CON). Behavior and physiologic outcome measures were collected pre-procedure and up to 48 h post-procedure. The results suggest that MEL and FLU were equally effective at providing post-operative analgesia; however, even with NSAID administration, acute pain and inflammation were still present in surgical sheep compared to non-surgical controls. The facial grimace scale results were not consistent with the other outcome measures taken in this study and it should not be used as a stand-alone pain assessment tool.

**Abstract:**

The amount of scientific data evaluating sheep pain responses after analgesia treatment is limited. The aims of this study were to compare the efficacy of flunixin meglumine (FLU) and meloxicam (MEL) at relieving post-surgical pain in sheep and to evaluate the utility of the Sheep Grimace Scale (SGS). Thirty ewes were assigned to one of three treatment groups: oral MEL or intravenous FLU to manage pain associated with a laparotomy procedure, or a non-surgical control (CON) group. Behavior and physiologic outcome measures were collected pre-procedure and up to 48 h post-procedure. There were no significant differences in behavior, gait, degree of inflammation or pain around the surgical site when MEL and FLU sheep were compared, suggesting that both drugs provided similar levels of analgesia. Significant differences in behavior, gait, abdominal inflammation and pain were found when surgical sheep were compared to non-surgical controls. More work is needed to characterize the amount of pain relief provided by MEL and FLU. The SGS had moderate reliability between scorers; however, the results were inconsistent with the other study outcome measures. The SGS may have some utility as a pain assessment tool but should be used in conjunction with other pain measures.

## 1. Introduction

Over five million sheep and lambs are raised each year in the United States for meat, wool and milk [1]. Throughout their production lives, animals may experience pain associated with common management practices and/or infectious diseases, such as mastitis and foot rot [2]. Dystocia, or a difficult birth characterized by prolonged delivery, with or without human assistance, is a common source of pain in ewes and a leading cause of perinatal lamb mortality [3,4,5,6]. Elective husbandry procedures, including surgical castration, tail docking and mulesing, along with shearing wounds are further sources of pain associated with sheep and lamb production [7,8]. However, sheep are not provided routine pain management for any of these conditions or procedures, which is a significant animal welfare concern. Producers and veterinarians cite treatment costs, lack of analgesic drugs licensed for use in sheep (currently, there are none approved in the U.S.), risk of drug residues in the tissues or milk and limited scientific data evaluating pain responses before and after treatment as reasons why pain management is not provided [3,9]. Sheep do not show obvious behavioral signs of pain, which can make pain assessment difficult and may be another contributing factor for inadequate pain management [10].

Flunixin meglumine is a non-steroidal anti-inflammatory drug (NSAID). Flunixin is the only analgesic approved to manage pain in an agriculture species (cattle; transdermal) by the U.S. Food and Drug Administration (FDA) [11]. Intravenous (IV) administration has demonstrated efficacy at reducing the pain and stress response of dehorning and surgical castration in calves [12,13] and improving lameness in steers and cows [14,15]. Flunixin has been used as a supportive treatment for ewes with clinical mastitis [16] and has effectively reduced surgical castration and tail docking pain in lambs [17].

Meloxicam is an NSAID commonly used extra-label to manage pain in livestock species in the U.S. Meloxicam has proven efficacy in reducing pain and inflammation associated with dehorning and castration of calves [18,19], in reducing lameness severity in calves [20] and sheep [21] and reducing pain in lambs after mulesing and tail docking [22]. Meloxicam is also approved for use in the alleviation of pain and inflammation in sheep in many countries, including Canada and Australia.

A novel tool for pain assessment in sheep is the Sheep Grimace Scale (SGS), which describes changes to facial features in response to pain [2,23]. A similar scale has been developed for lambs [24]. Grimace scales have allowed for the rapid detection of pain, leading to faster analgesic intervention and improved animal welfare [25,26]. Currently, the SGS has not been used in published studies by researchers uninvolved with its development. Therefore, it is difficult to determine the utility and external validity of the Sheep Grimace Scale as a pain assessment tool. 

The objectives of this study were (1) to compare the efficacy of flunixin meglumine (IV) and meloxicam (oral) at relieving post-surgical pain in sheep and (2) to evaluate the utility of the Sheep Grimace Scale as a pain assessment tool. We hypothesized that both NSAIDs would be effective at reducing pain, based on behavior and physiologic outcome measures, and that the SGS will be a useful tool to detect pain in sheep. 

## 2. Materials and Methods 

All animal use and procedures were approved by the Institutional Animal Care and Use Committee at Kansas State University prior to study commencement (Protocol #4315). 

### 2.1. Animals

Thirty ewes (10 Hampshire, 10 Rambouillet and 10 Polypay; BW = 72.1 ± 10.9 kg) were used in this pilot study. Sheep were housed individually in raised metal pens (1.5 × 2.4 m) with a grated floor at the Kansas State University Sheep and Meat Goat Center (Manhattan, KS, USA). They were fed daily rations of brome/prairie hay that met maintenance energy requirements and had ad libitum access to water for the duration of the study. Sheep were initially enrolled in a nutrition trial run by another group of researchers (Protocol #4305). As part of the nutrition study, a subset of the sheep (*n* = 12) were given a supplement containing a by-pass protein formulation with high energy and protein content (Soy Plus; Dairy Nutrition Plus, Ames, IA, USA) for 2 weeks. The goal of providing the supplement to ewes was to stimulate follicular development and increase ovulation. To assess the potential benefits of the supplement on ovulation at the end of the trial, ewes underwent a laparotomy to allow the researchers to measure follicle size and collect aspirated contents for further analysis. Sheep were enrolled in this study 24 h prior to the laparotomy procedure, to provide and assess the efficacy of post-operative analgesia to manage pain.

### 2.2. Treatments and Laparotomy Procedure

Twelve ewes were randomly assigned to each analgesia treatment group: meloxicam (MEL) or flunixin meglumine (FLU). Treatments were balanced across breed (*n* = 4 Hampshire, *n* = 4 Rambouillet and *n* = 4 Polypay per analgesia group; *n* = 24 sheep total) and supplement status (*n* = 6 supplement and *n* = 6 no supplement per analgesia group) from the nutrition trial. Six ewes served as study controls (CON) and were equally represented across the three breeds (*n* = 2 sheep per breed).

All ewes (including controls) were held off feed for 24 h and water for 12 h prior to sedation, to prevent regurgitation during surgery. Approximately 1 h prior to the laparotomy, 2.0 mg/kg oral MEL (Meloxicam Tablets, USP 15 mg; Zydus Pharmaceuticals Inc., Pennington, NJ, USA) or 2.2 mg/kg FLU IV (Banamine 50 mg/mL; Zoetis Inc., Parsippany, NJ, USA) was administered to ewes, according to their treatment group. Meloxicam tablets were first dissolved in water and then administered to sheep using a syringe. At the time of the procedure, sheep were individually transferred to a holding pen and sedated by the other team of researchers with 0.2 mg/kg midazolam (Midazolam Injection, USP 5 mg/mL; West-Ward Pharmaceuticals Corp., Tinton Falls, NJ, USA), 4.0 mg/kg ketamine (Zetamine 100 mg/mL; MWI Animal Health, Boise, ID, USA) and 0.1 mg/kg butorphanol (Torbugesic 10 mg/mL; Zoetis Inc., Parsippany, NJ, USA), all administered IV. Sedated sheep were placed in dorsal recumbency and carried to a nearby surgical suite. Their ventral midline was clipped and aseptically prepared, and 10 mL of lidocaine HCl (2%; MWI Animal Health, Boise, ID, USA) was administered subcutaneously. A 10 cm incision was then made through the skin, subcutaneous tissues and linea alba. The uterus and both ovaries were exteriorized for ovarian follicular analysis and aspiration. The surgical procedure took approximately 30 min.

After data collection associated with the nutrition trial (necessitating sheep undergo a laparotomy) was completed, the incision was sutured closed and sheep were removed from the surgical suite. They were placed in sternal recumbency in a recovery pen, bedded with straw, and monitored. Once ewes were fully recovered from sedation, standing and mobile, they were returned to their individual pens. CON sheep were sedated only in their individual pens with 0.2 mg/kg midazolam IV and 0.1 mg/kg butorphanol IV and did not undergo a surgical procedure. They were monitored until fully recovered. All ewes in this study recovered from sedation without incident. The ventral midline of CON sheep was clipped 24 h prior to study commencement. 

At 24 and 48 h post-procedure, sheep in the MEL group received 1.0 mg/kg meloxicam orally and sheep in the FLU group received 2.2 mg/kg flunixin IV. The pharmacokinetics of both flunixin meglumine IV and oral meloxicam have been described in sheep [27,28] which allowed for the determination of appropriate drug administration regimens in this study.

### 2.3. Outcome Measures

Outcome measures were collected in the following order at each time point: behavior, blood collection, infrared thermography, mechanical nociceptive threshold, pressure mat gait analysis, vocalization and facial grimacing (the last three measures were collected simultaneously). At 24 and 48 h, NSAIDs were administered after blood collection.

#### 2.3.1. Behavior Recording and Scoring

Video cameras (Sony Handycam HDR-CX405, Sony Corporation of America, New York, NY, USA) were placed on tripods outside of each pen. Sheep were video recorded the day prior to the laparotomy procedure for 30 min, to collect baseline behavior data. Post-procedure, sheep were video recorded for 30 min at the following time points: 4, 6, 24, 30 and 48 h. The videos were randomized across time point and sheep ID using a random number generator (random.org). Each video was scored continuously by a trained observer blinded to treatment, time point and surgery status of the sheep using BORIS software (Behavioral Observation Research Interactive Software v 7.7.3, Torino, Italy) and a detailed ethogram (Table 1). The ethogram was derived from multiple pain assessment studies on sheep [29,30] and dairy cows [31]. The total duration of scored behaviors was converted into proportions of time prior to analysis to create time budgets. A total of 5400 min (90 h) of behavior recordings were scored and analyzed for this study. 

#### 2.3.2. Infrared Thermography (IRT) Imaging

Infrared thermography images of the surgical site (or the lower abdomen for CON sheep, in the area where the incision was made in MEL and FLU sheep) were collected from each ewe pre-procedure (baseline) and at 4, 6, 24, 30, and 48 h post-surgery using a research grade infrared thermography camera (FLUKE TiX580; FLUKE Corporation, Everett, WA, USA). The camera was calibrated to the ambient temperature and relative humidity of the room prior to taking images. One individual gently restrained each ewe in the standing position for approximately 30 s to facilitate image capture. Another individual held the IRT camera underneath the ewe, in-line with the surgical site at a distance of approximately 0.5 m, and collected one image. 

Infrared images were analyzed using research grade software (SmartView 4.3; FLUKE Corporation, Everett, WA, USA). For each image collected, the temperature at four sites around the incision (left cranial, left caudal, right cranial, right caudal) were recorded, along with the average temperature across the four sites. For the CON sheep, these four sites were estimated based on where the incision would have been made. These data were used to assess the degree of inflammation and compare the ability of MEL or FLU to reduce the inflammation associated with soft-tissue surgery in sheep. 

#### 2.3.3. Mechanical Nociceptive Threshold (MNT) Determination

The mechanical nociceptive threshold refers to the lowest amount of pressure an animal can tolerate before a behavioral response indicative of pain occurs [32]. The MNT was determined on the lower abdomen of sheep pre-procedure (baseline) and at 4, 6, 24, 30, and 48 h post-surgery around the site of incision (or the lower abdomen for CON sheep, in the area where the incision was made in MEL and FLU sheep) using a hand-held pressure algometer (Wagner Instruments, Greenwich, CT, USA). A withdrawal (pain) response in sheep was indicated by any overt movement away from the applied pressure algometer. Four sites around the incision (left cranial, left caudal, right cranial, right caudal) and one control site on the upper abdomen of the ewe, approximately 15 cm away from the incision, were measured at each time point. For the CON sheep, these four sites were estimated based on where the incision would have been made. The location of the test sites, the order of data collection from each test site, and the individual measuring MNT did not change for the duration of the study. 

#### 2.3.4. Blood Collection, Processing, and Drug Concentration Analysis

A blood sample (6.0 mL) from each ewe was obtained via direct venipuncture of the jugular vein at baseline and at 4, 6, 24, 30, and 48 h post-surgery. Blood was immediately transferred into an additive-free blood collection tube (BD Vacutainer; Franklin Lakes, NJ, USA) and stored on ice before processing. Blood samples were then centrifuged for 10 min at 1500 g. Collected plasma was placed in cryovials in duplicate with a single-use transfer pipette and frozen at −80 °C until analysis. 

Plasma drug concentrations for flunixin and meloxicam were determined using ultra-high pressure liquid chromatography coupled with mass spectrometry (UPLC-MS). For each drug, a standard curve ranging from 0.1 to 250 ng/mL with a correlation coefficient of at least 0.98 was used. 

Pharmacokinetic (PK) analysis was performed with a non-compartmental approach using a commercially available software (Phoenix^®^ v 8.3; Certara Inc., Princeton, NJ, USA). Cmax and AUClast for individual animals were calculated, and the descriptive statistics (geometric mean, minimum, median, and maximum values) were summarized. One ewe in the FLU group was excluded from the PK analysis, as the concentration of flunixin were undetectable at 24 and 48 h post-surgery and close to the lower limit of quantitation at 30 h post-procedure. One ewe in the MEL group was also excluded from the PK analysis, as the concentration of meloxicam were undetectable at all time points.

#### 2.3.5. Pressure Mat Gait Analysis

A commercially available floor mat-based pressure/force measurement system (Strideway, Tekscan Inc., South Boston, MA, USA) was used to record and analyze the gait of each ewe. Sheep walked across the pressure mat pre-procedure (for baseline gait determination) and again at 4, 6, 24, 30, and 48 h post-surgery. Sheep were able to walk across the pressure mat multiple times at baseline prior to data collection to ensure they were comfortable with the walkway. Video synchronization was used to ensure consistent gait between and within sheep. Research grade software (Strideway v 7.7; Tekscan Inc., South Boston, MA, USA) was used to measure stance time (s), contact pressure (kg/cm^2^), impulse (kg × s), contact force (kg) and stride length (cm) [20] by an individual who was blinded to treatment, time point and surgery status of the sheep. 

#### 2.3.6. Vocalization Recording and Analysis

Vocalizations were recorded as sheep walked across the pressure mat using a high-quality stationary microphone (Uhuru UM-900 USB Condenser Microphone; Rlg Communication Ltd., Dubai, United Arab Emirates). The maximum frequency (Hz), amplitude (μ) and energy (dB) of vocalizations were quantified from the collected audio files using Raven Pro Software (v 1.5; Cornell Lab of Ornithology, Ithaca, NY, USA) by an individual who was blinded to treatment, time point and surgery status of the sheep.

#### 2.3.7. Facial Grimace Analysis

A high definition video camera (GoPro Hero7 4k; GoPro Inc., San Mateo, CA, USA) was placed at the end of the pressure mat walkway and recorded the sheep each time they walked across the mat. Still-images of each ewe’s face were pulled from the collected video using the Everio MediaBrowser 4 program (Pixela Corporation, Osaka, Japan). In instances where one clear image of the ewe’s face could not be captured from the video camera at the end of the walkway, the videos collected for behavior scoring at the corresponding time point were consulted and an attempt to capture a still-image of the ewe in her pen was made. In total, 175 images were collected: 90% of the images were taken as sheep walked across the pressure mat and 10% were taken when ewes were in their pen. Images were then randomized using a random number generator (random.org). 

**Figure 1:**
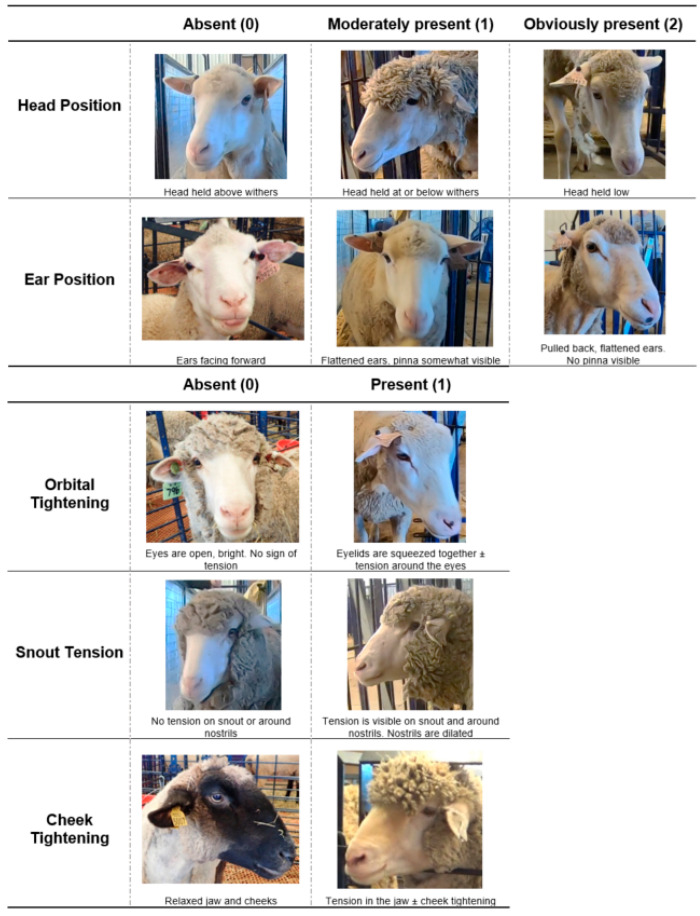
A modified Sheep Grimace Scale (SGS), incorporating facial action units described by McLennan et al. [2] and Häger et al. [23]. This SGS scores head position, ear position, orbital tightening, snout tension and cheek tightening. The maximum score is 7.

The Sheep Grimace Scale (SGS) was slightly modified for this study prior to scoring (Figure 1). This was done primarily to incorporate the facial action units defined in the two published Sheep Grimace Scales [2,23]. Orbital tightening was also moved to a 2-point scale (0–1; absent or present) as the intermediary or “moderately present” level was difficult to observe in the images from this study.

Three individuals blinded to treatment, time point and surgery status of the sheep used the SGS to score each image. The total SGS score for each image was calculated by summing the scores given to the five facial action units (head position, ear position, orbital tightening, snout tension and cheek tightening). Therefore, the minimum score possible was 0 and the maximum score possible was 7. The interobserver reliability of scoring each facial action unit was accessed by calculating the intraclass correlation coefficient (ICC) prior to statistical analysis. 

### 2.4. Statistical Analysis

Behavior results were analyzed using a generalized linear mixed model (GLIMMIX) with a beta distribution, including time, treatment, breed, supplement status (from the nutrition trial), and the time × treatment interaction in SAS (Statistical Analysis System 9.4, SAS Institute Inc., NC, USA). Time was a repeated measure with sheep as the experimental unit. Post hoc tests were conducted on significant factors using the Tukey–Kramer adjustment. Statistical significance was set at *p* < 0.05. 

All other outcomes (except for drug concentration analysis) were analyzed using a mixed model procedure in SAS, including time, treatment, breed, supplement status, and the time × treatment interaction. Time was a repeated measure with sheep as the experimental unit. A post-hoc Tukey’s test was conducted for significant outcomes. 

Potential differences of Cmax or AUClast between breeds and supplement status were analyzed with a non-parametric Kruskal–Wallis test for three groups or Mann–Whitney test for two groups using GraphPad Prism Version 9.0 (GraphPad Software, Inc., San Diego, CA, USA).

## 3. Results

### 3.1. Behavior

Two behaviors (eating: *p* = 0.007 and walking: *p* = 0.006) were affected by treatment across the observation period. There was also a trend in two additional behaviors having a treatment effect (chewing: *p* = 0.075 and sleeping: *p* = 0.088) (Table 2). Sheep in the CON group spent significantly less time eating throughout the observation period compared to MEL (*p* = 0.007) and FLU (*p* = 0.009) sheep. CON sheep also spent more time walking throughout the study compared to MEL (*p* = 0.006) and FLU (*p* = 0.003) sheep. There was a trend in CON sheep sleeping more than MEL sheep (*p* = 0.088). CON sheep also tended to chew on their body, substrates in their pen or conspecifics in nearby pens more than MEL (*p* = 0.070) and FLU (*p* = 0.073) sheep; however, this trend disappeared after the Tukey–Kramer adjustment.

There was a significant time effect for two behaviors (eating: *p* = 0.021 and walking: *p* = 0.009). Across the observation period, sheep spent significantly more time eating at 6 h post-surgery compared to the baseline, 4, 24, and 48 h time points (*p* < 0.05). Sheep also walked significantly more at 4 h post-procedure compared to 24 and 48 h (*p* < 0.05). 

Two behaviors (eating: *p* = 0.069 and lying: *p* = 0.073) tended to be more frequently observed in Hampshires compared to Polypays. There were no significant behavioral breed differences found in this study. 

Feeding behavior was significantly affected by the supplement status of sheep, with sheep who were provided the supplement as part of the nutrition study spending more time eating than non-supplement fed sheep (*p* = 0.034). There were no other behavior changes associated with supplement status found.

### 3.2. Infrared Thermography

**Figure 2:**
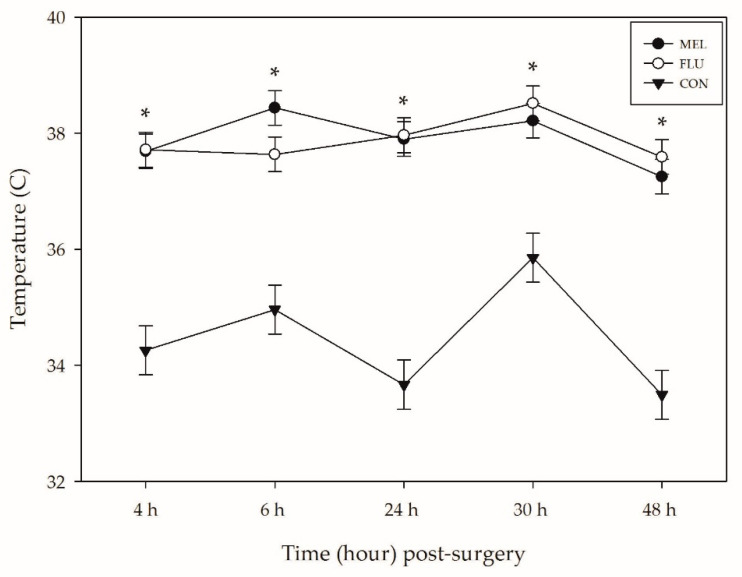
Mean infrared thermography temperature (°C ± SEM) of the tissues around the abdominal incision for each treatment group over time (*n* = 12 sheep per analgesia group; *n* = 6 controls). MEL = 2.0 mg/kg oral meloxicam pre-procedure and 1.0 mg/kg oral meloxicam at 24 and 48 h post-surgery; FLU = 2.2 mg/kg flunixin meglumine IV pre-procedure, at 24 and 48 h post-surgery; CON = non-surgical (control) group. Asterisk represents a significant difference (*p* < 0.05) between the sheep who underwent a laparotomy (MEL + FLU; *n* = 24) and the CON group.

The average temperature at the surgical site on the lower abdomen of sheep differed significantly across treatments (*p* < 0.0001), time (*p* < 0.0001), and breed (*p* = 0.0007). CON sheep had significantly lower abdominal temperatures across the observation period (34.3 ± 0.19 °C) compared to MEL (37.7 ± 0.12 °C; *p* < 0.0001) and FLU (37.7 ± 0.12 °C; *p* < 0.0001) sheep (Table 3; Figure 2). There was no significant difference in temperature between MEL and FLU sheep at any point in this study.

The average abdominal temperature of sheep at baseline was 35.5 ± 0.21 °C. At all of the post-surgical time points (4, 6, 24, 30, and 48 h), sheep had significantly higher surgical site temperatures compared to baseline (*p* < 0.01). Rambouillet sheep also had significantly higher surgical site temperatures across the observation period (37.0 ± 0.14 °C) compared to the Hampshires (36.4 ± 0.14 °C; *p* = 0.005) and Polypays (36.3 ± 0.14 °C; *p* = 0.002).

### 3.3. Mechanical Nociceptive Threshold

**Figure 3:**
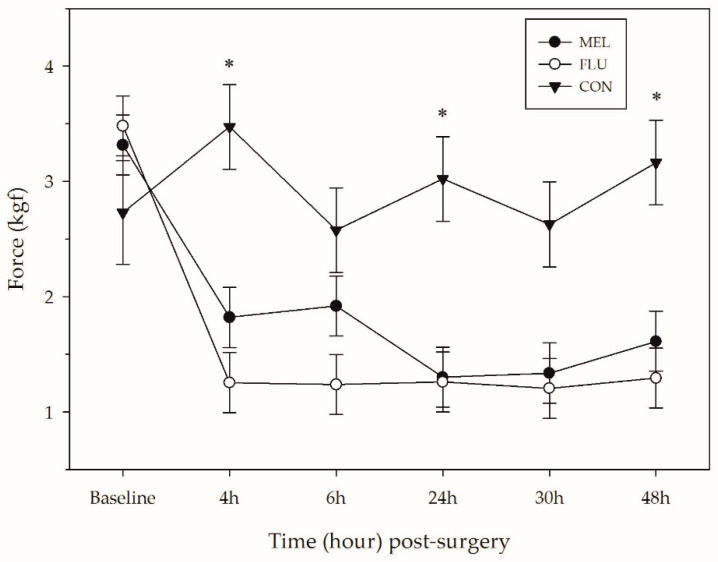
Mean mechanical nociceptive threshold (kgf ± SEM) around the abdominal incision for each treatment group over time (*n* = 12 sheep per analgesia group; *n* = 6 controls). MEL = 2.0 mg/kg oral meloxicam pre-procedure and 1.0 mg/kg oral meloxicam at 24 and 48 h post-surgery; FLU = 2.2 mg/kg flunixin meglumine IV pre-procedure, at 24 and 48 h post-surgery; CON = non-surgical (control) group. Asterisk represents a significant difference (*p* < 0.05) between the sheep who underwent a laparotomy (MEL + FLU; *n* = 24) and the CON group.

The average force tolerated around the surgical site of sheep differed significantly across treatments (*p* < 0.0001), time (*p* < 0.0001) and breed (*p =* 0.0001). There was also a time × treatment interaction found (*p* = 0.008). CON sheep were able to tolerate significantly more pressure across the observation period (average: 2.93 ± 0.16 kgf) compared to MEL (1.88 ± 0.11 kgf; *p* < 0.0001) and FLU (1.62 ± 0.11 kgf; *p* < 0.0001) sheep (Table 4; Figure 3). Pre-procedure, there was no significant difference in MNT of sheep found. At 4, 24, and 48 h post-procedure, CON sheep had significantly higher pressure tolerances than MEL and FLU sheep (*p* < 0.05). There was no significant difference in MNT between MEL and FLU sheep at any point in the study. 

The average force tolerated on the lower abdomen of sheep at baseline was 3.18 ± 0.19 kgf. At all of the post-surgical time points (4, 6, 24, 30, and 48 h), sheep tolerated significantly less pressure at the surgical site compared to baseline (*p* < 0.01). Hampshire sheep also had significantly lower MNTs across the observation period (1.74 ± 0.12 kgf) compared to the Polypays (2.42 ± 0.12 kgf; *p* = 0.0002) and Rambouillets (2.28 ± 0.12 kgf; *p* = 0.004).

### 3.4. Plasma Drug Concentration

The individual animal pharmacokinetic results of FLU or MEL plasma concentrations are presented in Table 5. The mean maximum concentration (Cmax) and standard deviation (SD) of FLU were 1415.06 ± 512.27 ng/mL (min–⁠max = 551.82–⁠2178.38 ng/mL), with a coefficient of variation (CV) of 36.2%. The FLU Cmax had a geometric mean of 1320.48 ng/mL and a median of 1389.14 ng/mL. The mean and SD of the area under the curve from the time of dosing to the last measurable (positive) concentration (AUClast) of FLU were 21,528.51 ± 8591.29 h × ng/mL (min–⁠max = 7721.23–⁠35,657.73 h × ng/mL) with a CV of 39.91%. The FLU AUClast had a geometric mean of 19,772.15 h × ng/mL and a median of 19,385.93 h × ng/mL.

The mean and SD of Cmax of MEL in this study was 2868.15 ± 871.37 ng/mL (min–⁠max = 1078.79–⁠4554.97 ng/mL) with a coefficient of variation (CV) of 30.4%. The MEL Cmax had a geometric mean of 2723.23 ng/mL and a median of 2749.14 ng/mL. The mean and SD of AUClast for MEL was 83,138 ± 25,577.7 h*ng/mL (min–⁠max = 19,418.3–⁠118,666.9 h*ng/mL) with a CV of 30.8%. The MEL AUClast had a geometric mean of 77,020.3 h*ng/mL and a median of 83,179.6 h*ng/mL.

There were no statistically significant differences in MEL Cmax and AUClast between breeds and supplement status of sheep in this study (*p* > 0.1). Likewise, there were no significant differences in FLU Cmax and AUClast between sheep breeds and supplement status (*p* > 0.05).

### 3.5. Pressure Mat Gait Analysis

**Figure 4:**
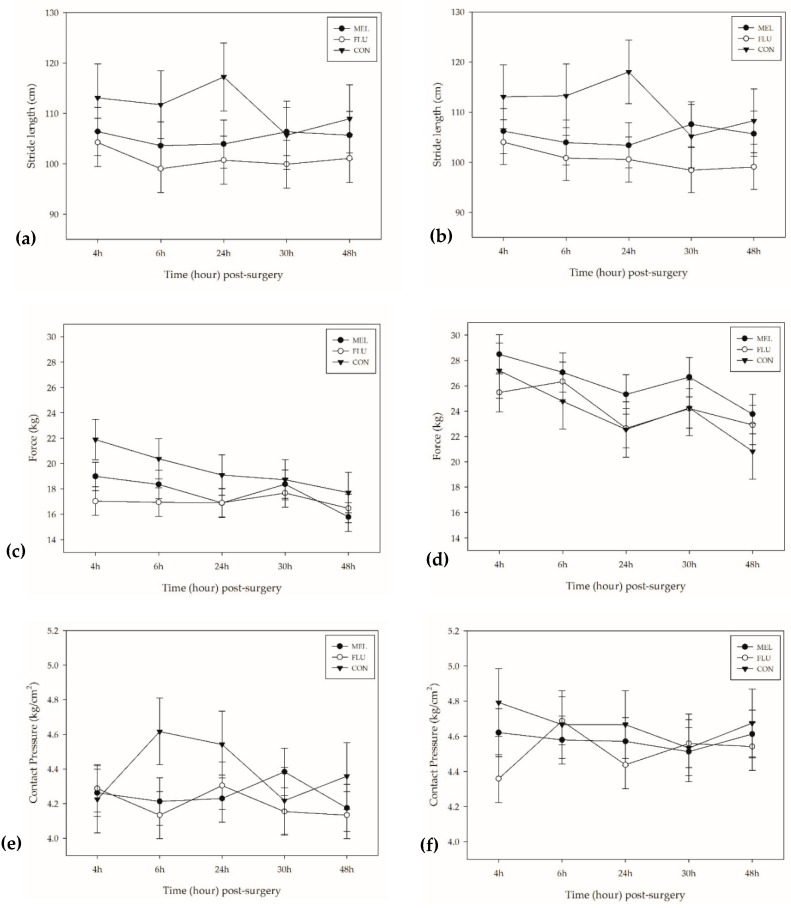
Mean (±SEM) over time for the following: (**a**) hind limbs stride length (cm); (**b**) front limbs stride length (cm); (**c**) hind limbs force (kg); (**d**) front limbs force (kg); (**e**) hind limbs contact pressure (kg/cm^2^); (**f**) front limbs contact pressure (kg/cm^2^). MEL = 2.0 mg/kg oral meloxicam pre-procedure and 1.0 mg/kg oral meloxicam at 24 and 48 h post-surgery (*n* = 12 sheep); FLU = 2.2 mg/kg flunixin meglumine IV pre-procedure, at 24 and 48 h post-surgery (*n* = 12 sheep); CON = non-surgical (control) group (*n* = 6 sheep).

The pressure mat gait analysis results are presented in Table 6 and Figure 4. 

#### 3.5.1. Stance Time

There were no treatment, time, or breed differences in stance time of sheep throughout the study (*p* > 0.1). The mean stance times for the hind limbs of MEL, FLU, and CON sheep were 0.34 ± 0.02 s, 0.36 ± 0.02 s, and 0.36 ± 0.03 s, respectively. The mean stance times for the front limbs of MEL, FLU, and CON sheep were 0.33 ± 0.02 s, 0.35 ± 0.02 s, and 0.31 ± 0.02 s, respectively.

#### 3.5.2. Stride Length

Stride length is the distance measured between the posterior heel of two consecutive foot falls. The mean stride length in both front and hind limbs of sheep differed significantly across treatment groups (*p* = 0.001 and *p* = 0.002, respectively). CON sheep took significantly longer strides using their front limbs (average: 112.18 ± 2.6 cm) compared to MEL (104.58 ± 1.8 cm; *p* = 0.045) and FLU (100.19 ± 1.8 cm; *p* = 0.0006) sheep. CON sheep also took significantly longer strides using their hind limbs (111.87 ± 2.7 cm) compared to FLU sheep (100.06 ± 1.9 cm; *p* = 0.002), with a trend in significance observed in MEL sheep (104.86 ± 1.9 cm; *p* = 0.09). There were no significant time or breed differences found.

#### 3.5.3. Force

Force refers to the maximum force applied to the mat for each step. There were significant time and breed effects found in the mean force applied by the front limbs of sheep (*p* = 0.013 and *p* = 0.007, respectively). Across all treatment groups, there was significantly more force applied by the front limbs of sheep at 4 h post-surgery (27.04 ± 1.0 kg) than at 48 h post-surgery (22.85 ± 1.0 kg; *p* = 0.036). Polypays had significantly lower measured force in their front limbs (22.97 ± 0.71 kg) compared to Rambouillets (26.03 ± 0.71 kg; *p* = 0.007), with a trend in significance observed in Hampshires (25.26 ± 0.71 kg; *p* = 0.056). 

In the hind limbs, there was a significant difference found in the amount of force applied between treatment groups (*p* < 0.0001), with CON sheep applying more measured force in their hind limbs (20.25 ± 0.65 kg) compared to MEL (17.47 ± 0.46 kg; *p* = 0.002) and FLU (16.68 ± 0.46 kg; *p* < 0.0001) sheep. 

#### 3.5.4. Impulse

Impulse refers to the maximum force applied per unit time. There was a significant breed effect found in the mean impulse in the front limbs of sheep (*p* = 0.021). Rambouillets had a significantly higher measured impulse in their front limbs (5.94 ± 0.28 kg × s) compared to Hampshires (4.97 ± 0.28 kg × s; *p* = 0.034), with a trend in significance observed in Polypays (5.04 ± 0.28 kg × s; *p* = 0.053). There were no significant time or treatment differences in impulse found in the front or hind limbs of sheep in this study. 

#### 3.5.5. Contact Pressure

Contract pressure refers to the peak amount of pressure applied by each foot fall on the mat. There were no significant treatment, time, or breed differences found in contact pressure of the front limbs of sheep; however, there were significant treatment and breed differences found in the hind limbs (*p* = 0.002 and *p* = 0.0007, respectively). CON sheep had significantly higher measured contact pressure across the observation period (4.53 ± 0.08 kg/cm^2^) compared to MEL (4.25 ± 0.06 kg/cm^2^; *p* = 0.012) and FLU sheep (4.18 ± 0.06 kg/cm^2^; *p* = 0.002). Rambouillets had significantly lower measured contact pressure in their hind limbs (4.15 ± 0.06 kg/cm^2^) compared to Hampshires (4.50 ± 0.06 kg/cm^2^; *p* = 0.0004).

### 3.6. Vocalization

There were no significant treatment, time, or breed differences in maximum frequency, amplitude, or energy of vocalizations emitted by sheep in this study (*p* > 0.05). There was only a trend in FLU sheep emitting vocalizations of lower energy (5.74 ± 2.82 dB) compared to MEL sheep (14.29 ± 3.54 dB; *p* = 0.053) across the observation period. 

### 3.7. Facial Grimacing

The ICC between the three observers for each of the facial action units (head position, ear position, orbital tightening, snout tension, and cheek tightening) was 0.77, 0.73, 0.50, 0.40, and 0.33, respectively. Observers had good reliability with scoring head position, moderate reliability with scoring ear position and had poor reliability with scoring orbital tightening, snout tension and cheek tightening [33]. Therefore, only head and ear positions were used in the analysis of sheep grimace scores in this study.

There were no significant treatment, time or breed differences found in sheep facial grimacing in this study (*p* > 0.05). There was only a trend in MEL sheep grimacing more (average score: 0.70 ± 0.06) than CON sheep (0.46 ± 0.08; *p* = 0.057) throughout the observation period.

## 4. Discussion

This study compared the ability of meloxicam and flunixin meglumine to reduce pain associated with soft-tissue surgery in sheep. Irrespective of analgesia group, sheep who underwent the laparotomy procedure spent less time walking throughout the observation period compared to control sheep. Animals often show a decrease in general activity level or a reluctance to move when in pain [34,35,36]. However, other common behavior changes associated with pain in sheep, such as altered social interactions, postural changes to avoid contact with the source of pain, licking, rubbing or scratching the painful area, and reduced feed intake and rumination [37] were not observed. Conversely, both MEL and FLU sheep demonstrated an increase in feeding behavior in this study compared to non-surgical controls. This suggests that MEL and FLU may have been able to provide some pain relief to sheep; however, conclusions regarding analgesia efficacy based on behavior alone should be made cautiously. With sheep being a stoic species by nature, they often do not show overt behavioral signs of pain or distress, even when experiencing significant trauma or disease [36,38]. Because of this, pain behavior observations are challenging to collect and interpret consistently across studies [39]. This highlights the importance of assessing both behavior and physiologic changes associated with pain, especially in stoic or prey species, for a more rigorous approach to animal pain assessment. 

Infrared thermography is a non-invasive, validated tool to measure cutaneous temperature and assess inflammation [40,41]. Infrared thermography has been used to detect subclinical mastitis, fever, foot lesions and hoof infection in sheep [42,43,44,45]. Significant temperature differences were noted between sheep who underwent the laparotomy procedure and the non-surgical controls in this study, with greater temperatures of the lower abdomen (i.e., site of incision) recorded in MEL and FLU sheep at all post-surgical time points compared to CON sheep. This suggests that inflammation associated with the laparotomy procedure in sheep persisted beyond 48 h post-surgery, even when an anti-inflammatory drug was administered. Additionally, neither MEL nor FLU was found to be more effective at reducing this inflammation. A previous study assessed abdominal wound healing after a laparotomy in ewes, when 3.0 mg/kg ketoprofen (an NSAID) and 1.0 mg/kg pheniramine maleate (an anti-histamine) were provided to decrease inflammation post-procedure [46]. Even with drug administration, significant swelling of the surgical site was observed in ewes up to 72 h post-operatively [46], which is consistent with the results of this study. There may also be breed differences in susceptibility to post-surgical swelling. In this study, Rambouillet sheep had more inflammation at the site of incision than Polypays and Hampshires. Genetic and strain differences in inflammatory response have been described in laboratory mice [47,48]; however, this is an area that requires further exploration in livestock species. 

An increased sensitivity to pain, or hyperalgesia, can be caused by tissue damage and/or inflammation. Hyperalgesia has been described in sheep post-laparotomy and identified in sheep suffering from foot rot and experiencing lameness [49,50]. Pain sensitivity is often measured in livestock species via a mechanical nociceptive threshold (MNT) test using a pressure algometer [51]. Analgesia administration has been shown to increase MNTs, thereby reducing pain and hyperalgesia (sheep: [50]; calves: [18,52]). In this study, neither MEL nor FLU were able to increase the MNT of sheep post-laparotomy. While this contradicts our study hypothesis, it does correspond well with the IRT results: sheep post-procedure had increased inflammation and therefore, increased pain sensitivity at the surgical site. At 6 and 30 h post-procedure, MEL and FLU sheep did not have MNTs significantly different than CON sheep; however, this appears to be a result of CON sheep having lower MNTs at those time points than MEL and FLU sheep having higher MNTs. A similar phenomenon, where MNTs decreased in non-painful animals over time, has previously been described [53,54]. This may be due to animals becoming accustomed to the test and learning to respond as soon as the stimulus is applied (decreasing the resulting MNT) or animals simply becoming agitated and restless at repeated testing and restraint [53,54]. In this study, the lower MNTs of CON sheep at the 6 and 30 h time points were likely a result of the latter. Individual variability in nociceptive thresholds may also be related to genetic or breed differences in pain tolerance [55,56]. Hampshire sheep were significantly more sensitive to the MNT test than Polypays and Rambouillets. This result suggests that Hampshires were either experiencing more hyperalgesia associated with the laparotomy procedure or that Polypays and Rambouillets are more stoic sheep breeds and better able to mask their pain response. Difference in sheep breed pain expression is an area that needs to be studied further to improve the accuracy and interpretation of pain assessments. 

In lame animals or those experiencing localized pain, the redistribution of their body weight away from the affected limb or site of pain and towards the unaffected limb(s) is readily detected using pressure mat technology (lame dairy steers: [14]; lame meat goats: [57]; calves post-castration: [58]). This validated tool has been used previously to characterize weight distribution and limb placement in clinically healthy ewes [59], in lame ewes [21,60], and has demonstrated utility in detecting analgesic drug effects [61]. In this study, sheep were easily trained to walk across the pressure mat and there were no issues with collecting a complete data set (i.e., two steps forward per foot) from each ewe. Both MEL and FLU sheep had a significantly shorter stride length post-laparotomy compared to CON sheep. MEL and FLU sheep also had significantly less force and lower mat contact pressure of their hind limbs compared to CON sheep. These findings are consistent with gait changes in cattle experiencing pain due to lameness or surgical castration [61,62]. There were no gait differences between MEL and FLU sheep, further supporting the conclusion that meloxicam and flunixin meglumine provided similar levels of post-operative analgesia. 

Facial grimace scales have been developed for many species, including mice, horses and piglets [63,64,65]. Two grimace scales currently exist for sheep [2,23]. The McLennan et al. [2] study used five trained observers (training session involved) to score sheep facial expressions based on five facial action units: orbital tightening, cheek tightening, ear position, lip/jaw profile and nostril position. The study used naturally occurring painful disease states (footrot and mastitis) to develop their pain scale [2]. The observers had high inter-rater reliability (ICC of 0.86) and were accurately able to detect pain in sheep [2]. The Häger et al. [23] study used six experienced observers (scorers were provided a handout and brief explanation of how to use the grimace scale) to score sheep facial expressions using three facial action units: orbital tightening, ear/head position and flehming. The study used a surgical procedure (tibia osteotomy of the right hind leg) as their pain model [23]. The observers had high inter-rater reliability (ICC of 0.92) with good accuracy at detecting pain in sheep [23]. Our study used three observers: all observers had minimal experience with pain detection in sheep and two observers had no experience with using facial grimace scales for animal pain assessment. A brief training session occurred and the five facial action units used in the SGS were thoroughly discussed. The overall ICC was 0.64, indicating moderate reliability between scorers; however, only head and ear positions were within the moderate-to-good reliability range (ICC of 0.73–0.77). The variability in scoring orbital tightening, snout tension and cheek tightening was likely due to observer inexperience and sheep breed differences (e.g., the dark faces of the Hampshires made cheek tightening more difficult to score compared to the light faced Polypays and the heavy wool around the eyes of Rambouillets made scoring orbital tightening problematic). The results of the SGS in this study found no significant difference in facial grimacing between MEL, FLU, or CON sheep. This would suggest that MEL and FLU were able to eliminate post-operative pain, as surgical animals did not grimace more than non-surgical controls; however, this is inconsistent with the other study outcome results. It is more likely that pain was not accurately detected using the SGS. Using a more experienced cohort of scorers, subjecting observers to a robust training session before scoring, or making improvements to the scale itself to increase usability may have yielded better study results. Until facial grimace scoring of sheep can be done consistently and accurately, the SGS should be used in conjunction with other pain assessment measures. 

Acute pain serves a biologic function to alert an animal to injury, causing protective behavioral changes in the animal to prevent further tissue damage and promote healing [66]. To improve on-farm animal welfare, it is important to treat acute pain so that it does not become chronic and lead to animal suffering. While MEL or FLU were not able to eliminate post-surgical pain in sheep, it is likely that they were able to provide some pain relief. It is difficult to determine whether the amount of pain relief provided by the NSAIDs was significant without having a “pain + no analgesia” group; however, it would have been unethical to have sheep undergo a laparotomy procedure and not provide them with post-operative analgesia. To be assured that pain is effectively being mitigated after a major surgical procedure, sheep may need to be administered a more potent class of analgesic drug (e.g., opioid) or employ a multi-modal analgesia approach; however, the practicality of administering a controlled drug in a farm setting is low. This is an area that needs further examination in sheep and all livestock species.

A limitation of this study is the relatively small sample size when sheep breed was included in our statistical model. The differences in pain response between the breeds of sheep are interesting; however, more work on a larger scale is needed to confirm these preliminary results. Another limitation of this work was the inability to quantify the amount of pain relief provided by the NSAIDs. Future studies with meloxicam and flunixin should consider using a different pain model for ewes (e.g., lameness), where the ability to include a “pain + no analgesia” group would not compromise animal welfare. While there are challenges with designing studies around naturally occurring diseases on-farm, such as foot rot or mastitis, demonstrating that an NSAID is effective at relieving pain associated with these common disease states would improve the clinical and on-farm applicability. These considerations are important to drive analgesic drug approval for sheep by the U.S. FDA.

## 5. Conclusions

Meloxicam and flunixin meglumine provided similar levels of post-operative analgesia to sheep in this study. However, even with NSAID administration, acute pain and inflammation were still present in surgical sheep compared to non-surgical controls. It is unlikely that post-surgical pain in sheep would be eliminated using an NSAID; if pain elimination is the goal, a more potent class of analgesic or a multi-modal approach may need to be considered. A Sheep Grimace Scale may have some utility as a pain assessment tool but should be used in conjunction with other pain measures.

## Figures and Tables

**Table 1:** Ethogram used to score sheep behavior, grouped into maintenance, locomotion and posture, oral and olfactory behavior, social interaction and pain behavior (adapted from studies with sheep [29,30] and dairy cows [31]).

Behavior	Description
Eating	Ingesting food provided at feed bunk
Drinking	Consuming water from bucket
Defecating	Passing fecal matter in standing or lying position
Urinating	Passing urine in standing or lying position
Scratching	Using head or rear hoof to scratch the body
Sleeping	Lying down, eyes closed
Ruminating	Regurgitating, chewing, and swalling food
Grooming	Licking or rubbing body or head against pen
Walking	Moving forward at a normal pace
Standing	Body weight supported by legs, no forward movement
Lying	Recumbent, body on ground
Kneeling	Body weight supported by front carpal joints and hind legs
Licking	Moving tongue over surfaces or adjacent pen mates
Chewing	Nibbling at substrates, body or conspecifics in nearby pens
Sniffing	Inhaling air close to object or adjacent pen mate
Playing	Running, trotting, galloping, or springing alone or with adjacent pen mate
Butting	Head-to-head or head-to-body contact with adjacent pen mate
Agonistic	Biting or fighting adjacent pen mate
Allo-grooming	Licking or rubbing body against adjacent pen mate
Restlessness	Repeated sitting, standing, or walking for short durations, unsettled
Lip licking	Running the tongue over lips outside of feeding event
Tail wagging	Tail movement from side to side (or up and down)
Attention to surgical site	Attention to abdomen or site of tissue trauma. May include licking, or attempts to lick, surgical site
Abnormal postures	Abnormal standing or walking (tucked abdomen, hind limbs apart and further back than normal, walking unsteady, falling) and abnormal lying position (abnormal ventral or lateral recumbency, hind legs partially or fully extended, dog sitting)

**Table 2:** Proportion of time sheep were engaged in specific behaviors (*n* = 12 sheep per analgesia group; *n* = 6 controls) post-surgery. Values represent the proportional means (± SEM).

Behavior ^1^	Treatment ^2^			*p*-Value
	MEL	FLU	CON	
Eating	0.41 ± 0.04 ^a^	0.42 ± 0.04 ^a^	0.15 ± 0.04 ^b^	0.007
Walking	0.03 ± 0.00 ^a^	0.03 ± 0.00 ^a^	0.06 ± 0.01 ^b^	0.006
Chewing	0.00 ± 0.00	0.00 ± 0.00	0.01 ± 0.00	0.075
Sleeping	0.11 ± 0.04	0.24 ± 0.06	0.54 ± 0.20	0.085

^1^ Only significant (*p* < 0.05) behavior variables and trends (*p* < 0.10) are presented.^2^ MEL = 2.0 mg/kg oral meloxicam pre-procedure and 1.0 mg/kg oral meloxicam at 24 and 48 h post-surgery; FLU = 2.2 mg/kg flunixin meglumine IV pre-procedure, at 24 and 48 h post-surgery; CON = non-surgical (control) group. ^a,b^ Values within a row with different superscripts differ significantly.

**Table 3:** Mean infrared thermography temperature (°C ± SEM) at the four test sites around the abdominal incision for each treatment group (*n* = 12 sheep per analgesia group; *n* = 6 controls). For controls, these sites were estimated based on where the incision would have been made.

Location Around Incision	Treatment ^1^			*p*-Values		
	MEL	FLU	CON	Treatment	Time	Breed
Left cranial	37.4 ± 0.13 ^a^	37.4 ± 0.13 ^a^	33.9 ± 0.20 ^b^	<0.0001	<0.0001	0.037
Left caudal	37.5 ± 0.14 ^a^	37.5 ± 0.14 ^a^	34.5 ± 0.21 ^b^	<0.0001	<0.0001	0.004
Right cranial	37.7 ± 0.14 ^a^	37.8 ± 0.14 ^a^	34.0 ± 0.21 ^b^	<0.0001	<0.0001	0.008
Right caudal	38.1 ± 0.15 ^a^	38.0 ± 0.15 ^a^	34.6 ± 0.23 ^b^	<0.0001	<0.0001	<0.0001

^1^ MEL = 2.0 mg/kg oral meloxicam pre-procedure and 1.0 mg/kg oral meloxicam at 24 and 48 h post-surgery; FLU = 2.2 mg/kg flunixin meglumine IV pre-procedure, at 24 and 48 h post-surgery; CON = non-surgical (control) group. ^a,b^ Values within a row with different superscripts differ significantly (*p* < 0.05).

**Table 4:** Mean mechanical nociceptive threshold (kgf ± SEM) at the four test sites around the abdominal incision and at the control site on the upper abdomen for each treatment group (*n* = 12 sheep per analgesia group; *n* = 6 controls). For controls, these sites were estimated based on where the incision would have been made.

Location Around Incision	Treatment ^1^			*p*-Values		
	MEL	FLU	CON	Treatment	Time	Breed
Left cranial	2.31 ± 0.16 ^a^	2.09 ± 0.16 ^a^	3.53 ± 0.23 ^b^	<0.0001	<0.0001	0.019
Left caudal	1.45 ± 0.12 ^a^	1.30 ± 0.12 ^a^	2.64 ± 0.18 ^b^	<0.0001	<0.0001	0.087
Right cranial	2.19 ± 0.14 ^a^	1.83 ± 0.14 ^a^	3.33 ± 0.20 ^b^	<0.0001	<0.0001	<0.0001
Right caudal	1.59 ± 0.13 ^a^	1.27 ± 0.13 ^a^	2.59 ± 0.19 ^b^	<0.0001	<0.0001	<0.0001
Control	4.18 ± 0.17 ^ab^	3.96 ± 0.17 ^a^	4.69 ± 0.25 ^b^	0.057	0.033	0.34

^1^ MEL = 2.0 mg/kg oral meloxicam pre-procedure and 1.0 mg/kg oral meloxicam at 24 and 48 h post-surgery; FLU = 2.2 mg/kg flunixin meglumine IV pre-procedure, at 24 and 48h post-surgery; CON = non-surgical (control) group. ^a,b^ Values within a row with different superscripts differ significantly (*p* < 0.05).

**Table 5:** Individual ewe pharmacokinetic results. Cmax and AUClast results represent the mean values of data collected at three post-surgical time points (24, 30, and 48 h). 5-Hydroxy MEL and 5-hydroxy FLU represent the mean metabolized residue of meloxicam and flunixin, respectively.

Animal ID	Treatment ^1^	Breed	Supplement ^2^	Cmax (ng/mL)	AUClast (h*ng/mL)	5-Hydroxy MEL (ng/mL)	5-Hydroxy FLU (ng/mL)
35	MEL	Polypay	Yes	2620.69	83,932.19	9.6	- ^5^
50	MEL	Polypay	No	2849.20	69,231.46	8.8	-
68	MEL	Polypay	Yes	. ^3^	. ^3^	. ^3^	-
251	MEL	Polypay	No	2360.47	83,179.59	11.6	-
5007	MEL	Hampshire	Yes	4554.97	118,666.92	11.0	-
5132	MEL	Hampshire	Yes	2695.42	78,918.53	12.0	-
6138	MEL	Hampshire	No	2398.40	78,306.33	15.3	-
14037	MEL	Hampshire	No	2749.14	79,579.11	7.5	-
AD1424	MEL	Rambouillet	No	1078.79	19,418.29	. ^3^	-
AE0680	MEL	Rambouillet	No	3612.69	102,216.84	11.5	-
AG0776	MEL	Rambouillet	Yes	3251.56	101,675.44	7.3	-
WY070	MEL	Rambouillet	Yes	3378.31	99,392.72	11.5	-
64	FLU	Polypay	Yes	1443.38	26,502.07	-	33.7
405	FLU	Polypay	No	. ^4^	. ^4^	-	. ^4^
437	FLU	Polypay	No	1265.30	19,385.93	-	14.6
467	FLU	Polypay	Yes	1219.66	17,597	-	20.7
5119	FLU	Hampshire	No	1615.28	26,397.69	-	15.2
6039	FLU	Hampshire	Yes	1872.32	26,265.53	-	29.2
6094	FLU	Hampshire	Yes	1042.41	15,254.54	-	31.7
14124	FLU	Hampshire	No	2139.63	35,657.73	-	26.2
AD1421	FLU	Rambouillet	No	2178.38	32,041.31	-	33.3
AD1426	FLU	Rambouillet	Yes	848.30	11,705.38	-	20.0
AD1439	FLU	Rambouillet	Yes	551.82	7721.23	-	22.1
AD1440	FLU	Rambouillet	No	1389.14	18,285.21	-	16.3

^1^ MEL = 2.0 mg/kg oral meloxicam pre-procedure and 1.0 mg/kg oral meloxicam at 24 and 48 h post-surgery; FLU = 2.2 mg/kg flunixin meglumine IV pre-procedure, at 24 and 48 h post-surgery. ^2^ A subset of ewes were provided a daily supplement (Soy Plus; Dairy Nutrition Plus) 2 weeks prior to being enrolled in this study. ^3,4^ Concentration of MEL^3^ or FLU^4^ was undetectable. ^5^ Dash indicates “not applicable”.

**Table 6:** Mean (±SEM) outcome measures from the pressure mat gait analysis (*n* = 12 sheep per analgesia group; *n* = 6 controls).

Parameter	Treatment ^1^			*p*-Values		
	MEL	FLU	CON	Treatment	Time	Breed
Front limbs						
Stance time (s)	0.33 ± 0.02	0.35 ± 0.02	0.31 ± 0.02	0.28	0.36	0.49
Stride length (cm)	104.58 ± 1.8 ^a^	100.19 ± 1.8 ^a^	112.18 ± 2.6 ^b^	0.001	0.93	0.96
Force (kg)	25.59 ± 0.64	23.82 ± 0.64	24.85 ± 0.90	0.15	0.013	0.007
Impulse (kg × s)	5.50 ± 0.25	5.48 ± 0.25	4.96 ± 0.35	0.40	0.90	0.021
Contact Pressure (kg/cm^2^)	4.56 ± 0.05	4.50 ± 0.05	4.71 ± 0.08	0.089	0.94	0.16
*Hind limbs*						
Stance time (s)	0.34 ± 0.02	0.36 ± 0.02	0.36 ± 0.03	0.71	0.43	0.38
Stride length (cm)	104.86 ± 1.9 ^ab^	100.06 ± 1.9 ^a^	111.87 ± 2.7 ^b^	0.002	0.92	0.88
Force (kg)	17.47 ± 0.46 ^a^	16.68 ± 0.46 ^a^	20.25 ± 0.65 ^b^	<0.0001	0.25	0.26
Impulse (kg × s)	3.71 ± 0.18	3.87 ± 0.18	4.13 ± 0.25	0.40	0.71	0.60
Contact pressure (kg/cm^2^)	4.25 ± 0.06 ^a^	4.18 ± 0.06 ^a^	4.53 ± 0.08 ^b^	0.002	0.78	0.0007

^1^ MEL = 2.0 mg/kg oral meloxicam pre-procedure and 1.0 mg/kg oral meloxicam at 24 and 48 h post-surgery; FLU = 2.2 mg/kg flunixin meglumine IV pre-procedure, at 24 and 48 h post-surgery; CON = non-surgical (control) group. ^a,b^ Values within a row with different superscripts differ significantly (*p* < 0.05).

## Data Availability

The data presented in this study are available on request from the corresponding author.
